# Acute and subacute postsurgical pain in women with breast cancer: incidence and associations with biopsychosocial predictors—a secondary analysis of a randomized controlled trial

**DOI:** 10.1097/PR9.0000000000001058

**Published:** 2023-01-10

**Authors:** Alice Munk, Henrik Børsting Jacobsen, Julie Schnur, Guy Montgomery, Silje Endresen Reme

**Affiliations:** a The Mind Body Lab, Department of Psychology, Faculty of Social Sciences, University of Oslo, Oslo, Norway; b Department of Pain Management and Research, Oslo University Hospital, Oslo, Norway; c Center of Behavioral Oncology, Department of Population Health Science and Policy, Icahn School of Medicine at Mount Sinai, New York, USA

**Keywords:** Postsurgical pain, Breast cancer, Biopsychosocial, Pain expectancy, Psychosocial factors, Social support

## Abstract

**Introduction:**

Women who undergo breast cancer surgery risk suffering from postsurgical pain long after their surgery. Still, research on postsurgical pain in the subacute phase has been neglected.

**Objective:**

This study aims to investigate the incidence, intensity, unpleasantness, and presurgical predictors of acute and subacute postsurgical pain after breast cancer surgery.

**Methods:**

The study used an observational design through secondary analyses of the control group in a randomized controlled trial. Data from 102 women undergoing breast cancer surgery were included. Levels of acute and subacute pain intensity and unpleasantness were measured using 100 mm Visual Analogue Scales on the day of surgery and 4 weeks postsurgery. Linear regression analyses were performed to identify presurgical biopsychosocial predictors of acute and subacute postsurgical pain.

**Results:**

Average levels of postsurgical pain intensity and unpleasantness were as follows: 22.7 mm for acute pain intensity, 19.0 mm for acute pain unpleasantness, 10.3 mm for subacute pain intensity, and 11.7 mm for subacute pain unpleasantness. Pain expectancy predicted acute pain intensity (R^2^ = 0.04, *p* = 0.047) and acute unpleasantness (R^2^ = 0.06, *p* = 0.02). Perceived social support inversely predicted acute pain unpleasantness (R^2^ = 0.04, *p* = 0.014).

**Conclusion:**

Mild and moderate acute pain intensity and unpleasantness are common after breast cancer surgery, whereas levels of subacute pain intensity and unpleasantness are low. Pain expectancy predicts acute postsurgical pain intensity and unpleasantness, whereas expected social support inversely predicts acute postsurgical pain unpleasantness.

## Abstract 2

Some women who undergo breast cancer surgery experience intense and unpleasant acute and subacute postsurgical pain. Presurgical pain expectancy significantly predicts postsurgical pain.

## 1. Introduction

Over 1 million women undergo breast cancer surgery every year.^25^ Many of these procedures are day-case surgeries with minimal hospitalization time, and consequently, most of the recovery process occurs at home. This situation places greater responsibility for pain management on patients and their social support systems. One important aspect of recovery is postsurgical pain which represents a major clinical concern. Postsurgical pain is defined as pain that develops or increases after a surgical procedure and is not better explained by another cause or preexisting pain condition.^37^ Postsurgical pain develops in stages where acute postsurgical pain (APSP) is measured between 0 and 7 days postsurgery, subacute postsurgical pain (SPSP) between 7 days and 3 months, and chronic postsurgical pain (CPSP) is that which continues beyond 3 months.^26^ Mismanaged pain during the acute and subacute periods is not only uncomfortable but also associated with an increased risk of CPSP.^1,35^ In a recent study on APSP after mastectomy,^31^ most women experienced APSP, and 68.9% of the sample reported pain of moderate or greater intensity. Some surgical patients are discharged despite ongoing pain, at times with insufficient analgesic medication.^43,48^ In some of these cases, the pain intensity increases during the acute and subacute postsurgical periods^1,2^ possibly through peripheral and central sensitization processes.^21^ Still, almost no data exist on the incidence, intensity, and unpleasantness of SPSP in women undergoing breast cancer surgery. In 1 study on postmastectomy pain, 57.7% had severe APSP and 22.1% experienced severe pain 1-month postsurgery.^12^ To address these challenges, transitional pain services are becoming more common. These services offer multimodal assessment and pain management in the subacute phase and show promising results in preventing CPSP.^19,45^ To optimize transitional pain services, more data are needed on the incidence and intensity of acute and subacute pain after surgery. There is a significant need for research investigating which patients are most likely to benefit from such services, ie, patients at risk for heightened postsurgical pain. Currently, criteria for referral to transitional pain services are predominately biomedical (eg, type of surgery and level of nerve damage),^45^ whereas there are only a few examples of psychosocial referral criteria (eg, depression and pain catastrophizing).^19^ These psychosocial referral criteria are most likely based on their relationship to APSP and CPSP, since almost no data exist on predictors of SPSP. A recent meta-analysis on predictors of postsurgical pain after breast cancer surgery indicated that psychological factors increase the risk of intense acute and subacute pain, although these results are based on a small number of studies.^26^ In this review and meta-analysis, only a single study was found investigating the association between pain catastrophizing and subacute pain. This study found that higher presurgical pain catastrophizing increased the risk of clinically meaningful pain 2 months after breast cancer surgery.^3^

A huge knowledge gap on the critical subacute period remains. One must turn to the more frequently investigated phenomena of APSP and CPSP, where a set of biopsychosocial risk and resilience factors have been established in women with breast cancer (eg, age, presurgical pain, anxiety, depression, pain catastrophizing, and optimism).^26,40,47^

This study aims to fill in a gap in the literature by investigating the incidence, intensity, and unpleasantness of acute and subacute pain after breast cancer surgery. In addition, we aim to identify biopsychosocial predictors of acute and SPSP to help determine which patients might be most at risk.

Based on previous prevalence estimates of postsurgical pain in women with breast cancer, we expect most women to experience postsurgical pain when assessed the same day as the surgery and around one-third to still experience pain 4 weeks later.

We hypothesize that presurgical chronic pain, sociodemographic, surgery-specific, and psychosocial factors predict pain intensity and pain unpleasantness in the acute and subacute phases after lumpectomy and mastectomy. Since APSP has previously been found to be a predictor of CPSP in women with breast cancer,^47^ we expect APSP to predict SPSP.

## 2. Methods and materials

### 2.1. Study design, participants, and procedure

This study used a prospective, observational design and conducted secondary analyses of data from the control group in a randomized controlled trial (RCT) (“PREVENT”, ClinicalTrials.gov ID: NCT04518085). The trial was approved by the regional ethics committee (REK; ID: 67725) and the local data protection officer. The study design of the PREVENT trial has been fully described and published in the protocol.^34^ In brief, PREVENT investigated the effects of presurgical and postsurgical psychological interventions to prevent pain and fatigue after breast cancer surgery. Two hundred five women between 18 and 70 years who were scheduled to receive breast cancer surgery at the Department of Breast and Endocrine Surgery, Oslo University Hospital, Norway, were enrolled in the RCT between the 16th of October 2020 and the fourth of March 2022. One participant withdrew from the study shortly after inclusion and requested her data be deleted. Exclusion criteria were insufficient Norwegian language skills, cognitive and psychiatric impairments, or other serious malignancies. Participants were randomized to either the intervention or the attention control group using a randomization tool in the research management program VieDoc. The control group included 102 participants who were provided with an attention control session. The attention control was delivered approximately 1 hour before surgery as a 13-minute mindfulness prerecorded audio recording, which patients listened to through headphones. In this audio recording, patients were invited to focus on their breath while counting inhales and exhales until reaching 10 and then starting over again. The counting is prompted throughout the rest of the session. In previous studies, mindfulness-based interventions have shown small effects on persistent pain after surgery,^29^ including in women with breast cancer.^8^ However, these studies included several sessions of mindfulness meditation with some involvement of a therapist. Therefore, it was considered that a single mindfulness recording formed an ethically sound attention control condition in the PREVENT trial. Patients randomized to the intervention group in the PREVENT trial received a 15-minute long hypnosis session delivered by a trained therapist approximately 1 hour before surgery.^34^

At the Department of Breast and Endocrine Surgery at Oslo University Hospital, the perioperative procedure for women with breast cancer starts with multidisciplinary assessment and diagnosis. Patients were contacted by a patient coordinator by phone on the same day that the multidisciplinary team determined the patient's diagnosis. Patients were requested to come back to the department for an ambulatory consultation. Patients were advised to bring a relative or other support person with them. The ambulatory consultation usually took place the next day or within a few days of the coordinator call, and the consultation was run by the breast surgeon and a nurse. All patients were advised to feel free to contact the nurse at any time before surgery and were encouraged to contact the surgeon at any time they needed. Importantly, patients were offered support for psychosocial stressors, which are common in the diagnosis or preop period (eg, how to share the diagnosis with children or family).

All patients underwent breast cancer surgery under total intravenous anesthesia using propofol and remifentanil. Laryngeal masks were used to secure the airway. Perioperative fentanyl was given intravenously to reduce postoperative pain. At the postoperative care unit, standard postoperative analgesic medications included fentanyl or oxycodone, depending on the amount of pain, as well as paracetamol. Patients were discharged primarily with paracetamol and etoricoxib, whereas they were discharged with tramadol or oxycodone for more invasive surgeries.

One week after the surgery, patients met with a nurse for a postsurgical consultation. Four weeks after surgery, patients were called back for a postsurgical consultation with the surgeon and a nurse. Written information and schedules for the postsurgical consultations were administered to the patients on the day of surgery to increase the predictability of the postsurgical trajectory.

### 2.2. Data collection procedure

Baseline and demographic variables were collected using VieDoc, where patients can answer the questionnaires from home using a computer, tablet, or smartphone. Patients responded to baseline questionnaires approximately 1 to 7 days before surgery.

The outcome measures were collected by the hospital nurses using a paper form of the 100 mm Visual Analogue Scale (VAS) both the day of surgery in the postanesthesia care unit before discharge and 4 weeks after surgery. The hospital nurses were blinded for the patients' intervention condition in the main study (active or control). The subacute assessment was timed to take place during a standard postsurgical follow-up at the hospital and to correspond with optimal subacute biomarker assessments conducted in the main PREVENT study. It should be noted that the outcomes in this study are snapshots of single time points in the acute and subacute postsurgical period and not a measure of the full acute (0–7 days) and subacute (7 days to 3 months) postsurgical trajectories to reduce patient burden.

Information about intraoperative factors was obtained from the patients' medical records.

### 2.3. Measures

#### 2.3.1. Outcome measures

Pain intensity and pain unpleasantness were measured using 100 mm VAS. The VAS is a continuous scale represented as a horizontal line precisely 100 mm long. Patients are asked to rate the extent of their symptoms by marking the line. The mark is measured and scored, ranging from 0 to 100. Measuring pain with VAS has shown excellent psychometric properties in surgical patients.^4,23^ Reviews on pain assessment recommend capturing both the sensory and affective dimensions of pain, ie, by measuring pain unpleasantness along with pain intensity.^11,13^

#### 2.3.2. Sociodemographic and surgical information

Patient demographic characteristics and intraoperative information, including age (18–70 years), surgery type (mastectomy vs lumpectomy), and axillary lymph node dissection (ALND) (yes or no), were extracted from the patient's medical records. In a previous meta-analysis, younger age and ALND were evaluated as predictors of CPSP in women with breast cancer.^47^

#### 2.3.3. Presurgical pain

Existence of a presurgical chronic pain condition was measured using a shortened version of the Widespread Pain Index (WPI).^49^ The WPI has been validated in Norwegian chronic pain patients.^14^ Usually, in the WPI, patients rate their pain in 19 separate areas across 5 regions of the body (upper left, lower left, upper right, lower right, and upper body). The index is scored by counting 1 point for each painful area. In this study, pain was only rated across the 5 overall body regions, not the subregions. A question about pain duration was added to distinguish pain due to diagnostic tests (eg, sentinel node biopsy) from presurgical chronic pain conditions by asking if the pain had persisted for at least 3 months. Ultimately, the presence of a presurgical chronic pain condition was scored as a binary variable (yes or no) if patients had experienced pain in 1 or more regions for at least 3 months.

#### 2.3.4. Anxiety and depression

Self-reported anxiety and depression were measured using the Hospital Anxiety and Depression Scale (HADS). The HADS is designed for patients with physical illness and focuses on cognitive and emotional symptoms of depression and anxiety while excluding somatic symptoms such as pain or fatigue. Respondents rate their symptoms on a 4-point Likert scale. Higher scores indicate more anxious and depressed mood. The score can be divided into 2 subscores, 1 for anxiety and 1 for depression. Hospital Anxiety and Depression Scale has shown high validity and reliability in multiple clinical populations.^5^

In recent meta-analyses, presurgical anxiety and depression were significantly associated with CPSP in noncancer populations^15^ and with acute, subacute, and CPSP in women with breast cancer.^26^

#### 2.3.5. Pain catastrophizing

Pain catastrophizing is a robust predictor of pain after breast cancer surgery.^26^ The scale measures components of helplessness, magnification, and rumination in response to pain.^44^ Patients rate all items on a 4-point Likert scale, where 0 represents “not at all” and 4 “all the time.”

In this study, we measured pain catastrophizing using a shorter, 4-item version of the Pain Catastrophizing Scale (PCS-4) to decrease respondent burden. The PCS-4 consists of items 3, 6, 8, and 11 of the full-length PCS. The PCS-4 has shown good content validity, construct convergent validity, and criterion validity in surgical^7^ and chronic pain patients,^46^ with similar associations to outcomes of disability and pain as the full-length scale.

#### 2.3.6. Optimism

The Life Orientation Test, revised version (LOT-R), was used to measure dispositional optimism. The items in LOT-R assess generalized positive and negative outcome expectancies about the future.^36^ In previous studies, optimism has been linked to less psychosocial distress in cancer patients^20^ and less pain sensitivity.^16^ The scale contains 10 items, including 4 filler items that are not included in the score. The respondents rate each item from 0 = strongly disagree to 4 = strongly agree. The LOT-R can be scored as a total composite score or as 2 separate subscores on optimism and pessimism. Only the total score is included in this study. A higher total score indicates higher dispositional optimism.

#### 2.3.7. Social support

Expected social support is operationalized using the conceptual framework of social relations by Due et al.^10^ The scale measures the emotional and practical support expected to be available to the patient by asking, “Will any of the following people help or support you in everyday life if you need it?” in relation to “partner,” “family,” “friends,” “colleagues,” “neighbors,” or “others,” respectively. Respondents rate each item on a 6-point Likert scale ranging from “1 = always” to “6 = never or have none.”^39^ The scale is scored by adding scores of individual items into a total score. In the data analyses for the current study, scores are reversed so that higher scores mean higher perceived social support.

#### 2.3.8. Pain expectancy

Pain response expectancies were assessed with the single question, “how much pain do you expect to have after the surgery” using an Numeric Rating Scale (NRS) ranging from 0 = no pain at all to 10 = worst possible pain. Pain response expectancies have been found to predict pain intensity after breast cancer surgery.^27,38^

### 2.4. Statistical analyses

All baseline characteristics, including demographic, clinical, and psychosocial information, were analyzed using descriptive statistical methods. Continuous variables were reported using means, standard deviations, and ranges, whereas categorical variables were reported as frequencies and percentages.

Paired samples *t* tests were used to evaluate differences in mean levels of acute pain intensity and subacute pain intensity and acute pain unpleasantness and subacute pain unpleasantness.

The relationships between perioperative biopsychosocial factors and the primary outcomes of acute and subacute pain intensity and unpleasantness were investigated using univariate linear regression analyses with bias-corrected and accelerated bootstrapping.

All analyses were performed in the IBM SPSS Statistics version 28.

## 3. Results

Our sample included 102 women undergoing breast cancer surgery. Their mean age at the time of inclusion was 53 years ranging from 26 to 70 years, with a standard deviation of 1.0. Of our sample, 30.1% had mastectomies, whereas the rest had breast-conserving procedures. Of mastectomies, 94% were unilateral and 84% had primary reconstruction. Around 15.5% of the women had full ALND. Almost half of the women (44.7%) were experiencing a chronic pain state which had persisted for more than 3 months in the time leading up to surgery. All perioperative patient characteristics are reported in Table 1.

**Table 1:** Baseline biopsychosocial characteristics.

	Mean (SD, range)/N (%)*
Sociodemographics	
Age	53.0 (1.0, 26–70)
Living alone (yes)	24 (23.3%)
Working/student/military service	77 (74.8%)
Not working	24 (23.3%)
Highest educational status	
Primary school (1–10 y)	2 (1.9%)
Secondary school or vocational (11–13 y)	23 (22.3%)
College degree (14–17 y)	43 (41.7%)
Higher university (>17 y)	34 (33.0%)
Clinical characteristics	
Body mass index (kg/m^2^)	26.0 (4.4, 16.4–43.1)
Smoking (yes)	4 (3.9%)
Lumpectomy	70 (68.0%)
Mastectomy	31 (30.1%)
Sentinel lymph node biopsy	79 (76.7%)
Axillary lymph node clearance	16 (15.5%)
Previous surgeries other than the breast (yes)	83 (80.6%)
Previous breast surgeries (yes)	20 (19.5%)
Psychosocial characteristics	
Presurgical chronic pain (>3 mo) (yes)	46 (44.7%)
Expected postsurgical pain (0–10)	5.0 (2.0, 0–10)
Pain catastrophizing (0–16)	4.1 (3.3, 0–13)
Dispositional optimism (0–24)	16.8 (4.1, 5–24)
Perceived social support (6–36)	25.5 (6.2, 11–36)
Anxiety (0–21)	7.2 (4.1, 0–19)
Depression (0–21)	3.6 (3.0, 0–14)

* Continuous variables are presented as mean (SD, range); categorical variables are presented as n (%).

### 3.1. Acute and subacute pain intensity and pain unpleasantness after breast cancer surgery

**Figure 1.**
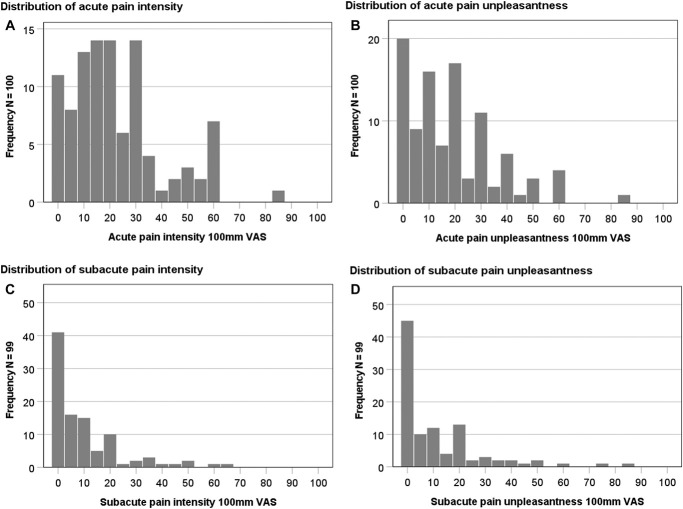
(A) Acute pain intensity after breast cancer surgery. (B) Acute pain unpleasantness after breast cancer surgery. (C) Subacute pain intensity after breast cancer surgery. (D) Subacute pain unpleasantness after breast cancer surgery.

The average level of acute pain intensity was 22.7 mm (range = 0–85 mm, SD = 18.0 mm) and 19.0 mm (range = 0–85 mm, SD = 17.3 mm) for pain unpleasantness on a 100 mm VAS scale. The distributions of the scores are illustrated in Figures 1A and B. Approximately 4 weeks later, women reported mean levels of subacute pain intensity at 10.3 mm (range = 0–65 mm, SD = 14.1 mm) and subacute pain unpleasantness at 11.7 mm (range 0–85 mm, SD = 16.7 mm) as illustrated in Figures 1C and D, respectively.

### 3.2. Predictors of acute and subacute pain intensity and pain unpleasantness

Table 2 presents biopsychosocial predictors of acute pain intensity and pain unpleasantness. Women who had presurgical expectations of higher pain experienced significantly higher pain intensity (F(1, 94) = 4.58, *p* = 0.047, adj. R^2^ = 0.04) and pain unpleasantness (F(1, 94) = 5.79, *p* = 0.022, adj. R^2^ = 0.06) immediately after waking up from surgery. Women who perceived themselves to have less social support experienced more acute pain unpleasantness (F(1, 93) = 5.22, *p* = 0.014, adj. R^2^ = 0.04). None of the remaining biopsychosocial variables predicted acute pain intensity or pain unpleasantness in this sample.

**Table 2:** Univariate linear regressions: predictors of acute pain intensity and pain unpleasantness.

	b	SE	β	*p*	BCa 95% CI's		Adj. R^2^
Predictors of acute pain intensity							
Age	−0.07	0.17	−0.04	*p* = 0.663	−0.38	0.24	−0.01
Axillary lymph node dissection	6.11	5.61	0.13	*p* = 0.274	−4.37	16.43	0.01
Lumpectomy vs mastectomy	5.36	3.97	0.14	*p* = 0.175	−2.59	13.61	0.01
Presurgical chronic pain	−0.23	3.60	−0.01	*p* = 0.961	−7.47	6.66	0.00
Pain expectancy	1.90	0.91	0.22	***p* = 0.047**	0.00	3.90	0.04
Optimism	−0.32	−0.35	−0.08	*p* = 0.370	−0.97	0.32	−0.01
Pain catastrophizing	0.87	0.56	0.17	*p* = 0.132	−0.26	1.88	0.02
Anxiety	0.59	0.37	0.14	*p* = 0.112	−0.08	1.28	0.01
Depression	0.32	0.49	0.01	*p* = 0.553	−0.65	1.32	−0.01
Social support	−0.43	0.25	−0.15	*p* = 0.088	−0.89	0.84	0.01
Predictors of acute pain unpleasantness							
Age	−0.09	0.15	−0.05	*p* = 0.562	−0.41	0.21	−0.01
Axillary lymph node dissection	3.63	4.42	0.08	*p* = 0.417	−5.18	13.67	0.00
Lumpectomy vs mastectomy	4.49	3.71	0.12	*p* = 0.235	−2.99	11.97	0.00
Presurgical chronic pain	1.86	3.45	0.05	*p* = 0.588	−4.66	8.36	−0.01
Pain expectancy	2.04	0.84	0.24	***p* = 0.022**	0.51	3.66	0.05
Optimism	−0.27	0.41	−0.06	*p* = 0.551	−1.09	0.55	−0.01
Pain catastrophizing	0.74	0.49	0.15	*p* = 0.127	−0.23	1.60	0.01
Anxiety	0.47	0.34	0.12	*p* = 0.171	−0.15	1.15	0.00
Depression	0.15	0.52	0.03	*p* = 0.770	−0.85	1.36	−0.01
Social support	−0.63	0.24	−0.23	***p* = 0.014**	−1.11	−0.17	0.04

Statistically significant results at *p* < 0.05 are written in bold adj. R^2^: adjusted R^2^, BCa 95% CIs: bias-corrected accelerated 95% confidence intervals based on 1000 bootstrap samples.

As shown in Table 3, none of the investigated biopsychosocial factors were significant predictors of subacute pain intensity or pain unpleasantness.

**Table 3:** Univariate linear regressions: predictors of subacute pain intensity and pain unpleasantness.

	b	SE	β	*p*	BCa 95% CIs		Adj. R^2^
Predictors of subacute pain intensity							
Age	−0.01	0.13	−0.07	*P* = 0.447	−0.35	0.13	−0.01
Axillary lymph node dissection	−0.64	3.75	−0.17	*P* = 0.857	−7.51	7.13	−0.01
Lumpectomy vs mastectomy	3.20	2.94	0.11	*P* = 0.290	−2.17	8.48	0.00
Presurgical chronic pain	3.31	2.84	0.12	*P* = 0.269	−2.30	9.06	0.00
Pain expectancy	0.75	0.63	0.11	*P* = 0.238	−0.47	1.83	0.00
Acute pain intensity*	0.22	0.12	0.26	*P* = 0.058	0.01	0.44	0.06
Optimism	−0.32	0.36	−0.09	*P* = 0.386	−1.13	0.40	0.00
Pain catastrophizing	0.65	0.43	0.15	*P* = 0.145	−0.11	1.53	0.01
Anxiety	0.47	0.33	0.14	*P* = 0.158	−0.17	1.20	0.01
Depression	−0.00	0.39	0.00	*P* = 0.999	−0.72	0.79	−0.01
Social support	−0.22	0.22	−0.09	*P* = 0.340	−0.65	0.19	−0.01
Predictors of subacute pain unpleasantness							
Age	−0.21	0.17	−0.13	*P* = 0.234	−0.55	0.08	0.01
Axillary lymph node dissection	4.65	4.65	0.10	*P* = 0.324	−3.10	11.81	0.00
Lumpectomy vs mastectomy	3.97	3.27	0.11	*P* = 0.230	−1.96	10.12	0.00
Presurgical chronic pain	2.90	3.31	0.09	*P* = 0.387	−3.45	9.76	0.00
Pain expectancy	0.96	0.70	0.11	*P* = 0.177	−0.46	2.24	0.00
Acute pain unpleasantness*	0.16	0.11	0.15	*P* = 0.159	−0.09	0.39	0.01
Optimism	−0.38	0.43	−0.09	*P* = 0.376	−1.23	0.46	0.00
Pain catastrophizing	0.65	0.50	0.13	*P* = 0.194	−0.23	1.61	0.01
Anxiety	0.63	0.41	0.16	*P* = 0.130	−0.22	1.55	0.01
Depression	0.47	0.44	0.09	*P* = 0.279	−0.38	1.30	0.00
Social support	−0.09	0.27	−0.03	*P* = 0.752	−0.59	0.38	−0.01

Statistically significant results at *p* < 0.05 are written in bold adj. R^2^: adjusted R^2^, BCa 95% CIs: bias corrected accelerated 95% confidence intervals based on 1000 bootstrap samples.

* Paired samples *t* tests showed that the differences in mean pain intensity and unpleasantness from the acute to the subacute phase were statistically significant at *p* < 0.001.

The *t* test comparing levels of pain intensity in the acute phase with levels of pain intensity in the subacute phase showed that the difference between acute pain intensity (M = 21.64 mm, SD = 16.52 mm) and subacute pain intensity (M = 10.13 mm, SD = 14.11 mm) was significant (t(97) = 6.06, *p* < 0.001, d = 0.61). Similarly, the difference of acute pain unpleasantness (M = 17.78 mm, SD = 15.51 mm) and subacute pain unpleasantness (M = 11.59 mm, SD = 16.59 mm) was also statistically significant (t(97) = 2.92, *p* = 0.004. d = 0.30).

## 4. Discussion

The aim of this study was twofold: to evaluate levels of pain intensity and pain unpleasantness in the acute and subacute phases after breast cancer surgery and to identify biopsychosocial predictors of acute and subacute pain intensity and pain unpleasantness.

Most women in our study reported what could be classified as mild APSP intensity and unpleasantness, ie, VAS scores between 1 and 30 mm,^18,41,42^ whereas some experienced moderate to severe symptoms. Similarly, in the subacute phase, most women were affected by mild pain intensity and unpleasantness and a considerable number of women experienced no pain or unpleasantness at all.

The women who expected higher pain going into surgery had higher pain intensity and pain unpleasantness in the acute postsurgical phase. Lower expected social support was a predictor of acute pain unpleasantness. The effect sizes were small but significant. Contrary to our hypotheses, none investigated biopsychosocial factors predicted subacute pain intensity or unpleasantness.

A previous study investigated the APSP trajectory after breast cancer surgery and found that 1 day after breast cancer surgery, 46.3% of women experienced no or mild pain, 33.3% moderate pain, and 20.3% severe pain.^30^ Here, patients self-reported APSP on the 0 to 10 NRS, and cut-offs were defined as no or mild pain ranging from 0 to 3, moderate pain 4 to 6, and severe pain 7 to 10 on the NRS. In our study, APSP was assessed on the same day as the surgery after waking up at the postanesthesia care unit before discharge. During this time, patients are most likely still affected by anesthesia which may have resulted in lower pain scores. Very little data exist on the incidence, intensity, and unpleasantness of SPSP. A previous study reported that 15% of women who had undergone breast cancer surgery reported clinically meaningful SPSP.^3^ Clinically meaningful pain was defined as the average pain intensity during the past 24hours ≥3 of 10 on the Brief Pain Inventory^9^ and was assessed 2 months postsurgery. Comparing these results with ours, some methodological differences, such as assessment timing and instrument differences, should be taken into consideration. To date, no universal cut-offs exist for the severity of VAS pain intensity or unpleasantness, nor specific cut-offs for patients undergoing breast cancer surgery. Overall, our results indicate that postsurgical pain in our sample is well managed. The comprehensive information and care which are standard procedures for breast cancer surgery at the Oslo University Hospital may explain why levels of postsurgical pain and unpleasantness are low in our sample. For example, presurgical consultations with the surgeon and nurse last 1 to 1.5 hours to ensure adequate time for patients to feel safe and well-prepared for surgery. In addition, nurses offer patients psychosocial support before and after surgery. Not all institutions are able to offer similar time and recourses, which could be one reason why levels of postsurgical pain following breast cancer are more severe in other studies.

When trying to predict which patients may need closer follow-up after hospital discharge, the identification of biopsychosocial risk and resilience factors is crucial. When reviewing the empirical background, each biopsychosocial predictor showed a consistent relationship to APSP. Taken together, one meta-analysis including both noncancer and cancer populations,^40^ and one focusing on women undergoing breast cancer surgery,^26^ conclude that presurgical pain catastrophizing, anxiety, depression, pain expectancy, and optimism all have weak but significant associations to APSP. Biological factors such as younger age, presurgical pain, and ALND have been identified as risk factors for APSP,^6,33^ and CPSP,^47^ where ALND, in particular, was found to have a moderate to large effect. It is, therefore, surprising that our results do not find a relationship between any of the biological factors and postsurgical pain. From a clinical perspective, presurgical pain expectancy is interesting since it is a modifiable risk factor. A meta-analysis concluded that expectancy interventions have moderate to large analgesic effects on acute clinical pain.^32^ Social support has previously been linked to pain through behavioral and neuroendocrine mechanisms.^22^ Our findings on the relationship between social support and pain unpleasantness were surprising since recovery in the postdischarge period was expected to depend more on levels of social support from friends and family members than in the acute phase while still being at the hospital. Our results may be better explained as another example of an expectancy effect: women who expect to receive social support may be less stressed and have lower levels of inflammation and pain sensitivity^28^ whether or not they call on that support. Inflammation and pain have previously been suggested as a cluster with low social support along with depression in women with breast cancer.^17^

Few previous studies have looked at associations between biopsychosocial factors and SPSP. These have indicated small effects of anxiety, depression, and pain catastrophizing on SPSP.^26^ Each biopsychosocial factor has been associated with developing CPSP in noncancer populations^15^ and women with breast cancer.^24,26,47^ This indicates that the biopsychosocial correlates of pain may vary throughout the postsurgical trajectory.

## 5. Limitations and future directions

In this study, the acute and subacute pain outcomes were each assessed at single time points. Future research should consider using more frequent assessments to evaluate the postsurgical pain trajectory more thoroughly.

The relatively small sample size in this study could obfuscate small effects. Future research should investigate these associations in larger samples resulting in more statistical power and the ability to fit more complex statistical models. Looking at interactive, cumulative, and nonlinear effects of biopsychosocial risk and resilience factors for pain would be a more accurate model of the complex, cyclic, and multifaceted phenomenon of pain. Regarding generalizability, our sample seems to have performed remarkably well for having low postsurgical pain and unpleasantness and low levels of psychosocial risk factors. Since this is a secondary analysis of an RCT, all participants signed up for an intervention study which could indicate they are healthier and more committed to improving their recovery than the general population. Although previous literature has not indicated any notable effects of a single presurgical mindfulness session, control group participants did get a relaxing intervention after all which could have helped in some way to manage their pain. For future research, it could be relevant to repeat the study in a strictly observational design.

## 6. Conclusion

Mild and in some cases moderate pain is common in the acute phase after breast cancer surgery. A snapshot of SPSP four weeks after breast cancer surgery showed that most women in our sample experience mild pain and unpleasantness, and many have no pain at all. Presurgical pain expectancy predicted acute pain intensity and unpleasantness, and lower expected social support was an additional predictor of acute pain unpleasantness. Our findings imply that clinicians should address patients' pain expectancies before surgery and assess for perceived social support. Patients' negative pain expectancies could be modified using psychosocial interventions to decrease postsurgical pain intensity and unpleasantness.

## Disclosures

The authors have no conflicts of interest to declare.

The randomized controlled trial that was basis for these secondary analyses was funded by the Norwegian Cancer Society (201906-2019). The funding sources do not have any role in the design of the study, data collection, analysis and interpretation of data, or decisions
